# Morphogenesis and oxygen dynamics in phototrophic biofilms growing across a gradient of hydraulic conditions

**DOI:** 10.1016/j.isci.2021.102067

**Published:** 2021-01-20

**Authors:** Anna Depetris, Hannes Peter, Ankur Deep Bordoloi, Hippolyte Bernard, Amin Niayifar, Michael Kühl, Pietro de Anna, Tom Jan Battin

**Affiliations:** 1 Stream Biofilm and Ecosystem Research Laboratory, School of Architecture, Civil and Environmental Engineering, École polytechnique fédérale de Lausanne, 1015 Lausanne, Switzerland; 2 Institute of Earth Sciences, University of Lausanne, 1015 Lausanne, Switzerland; 3 Marine Biological Section, Department of Biology, University of Copenhagen, Strandpromenaden 5, 3000 Helsingør, Denmark

**Keywords:** Microbiofilms, biophysics

## Abstract

Biofilms are surface-attached and matrix-enclosed microbial communities that dominate microbial life in numerous ecosystems. Using flumes and automated optical coherence tomography, we studied the morphogenesis of phototrophic biofilms along a gradient of hydraulic conditions. Compact and coalescent biofilms formed under elevated bed shear stress, whereas protruding clusters separated by troughs formed under reduced shear stress. This morphological differentiation did not linearly follow the hydraulic gradient, but a break point emerged around a shear stress of ~0.08 Pa. While community composition did not differ between high and low shear environments, our results suggest that the morphological differentiation was linked to biomass displacement and reciprocal interactions between the biofilm structure and hydraulics. Mapping oxygen concentrations within and around biofilm structures, we provide empirical evidence for biofilm-induced alterations of oxygen mass transfer. Our findings suggest that architectural plasticity, efficient mass transfer, and resistance to shear stress contribute to the success of phototrophic biofilms.

## Highlights

• We explored the structural differentiation of phototrophic biofilms in flume experiments
• Two distinct morphologies emerged depending on bed shear stress
• Oxygen measurements suggest efficient mass transfer in both morphologies
• Feedbacks between structure and hydraulics drive biofilm morphogenesis

## Graphical abstract

**Figure:**
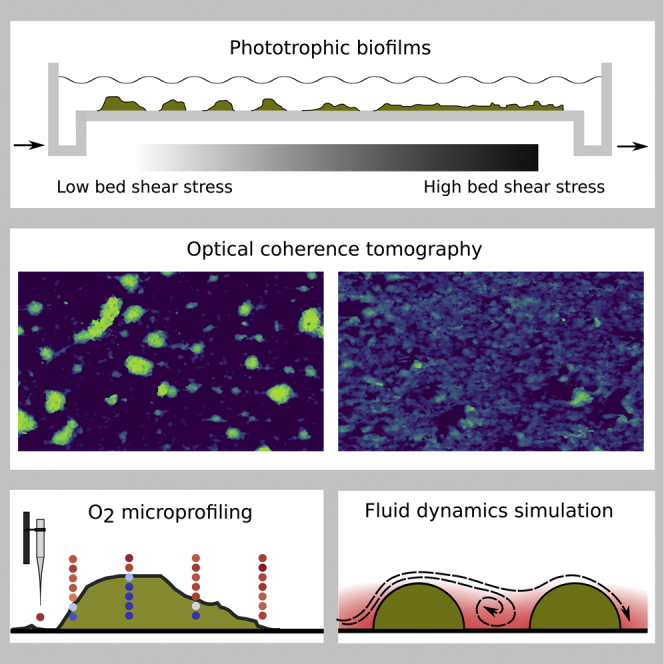

## Introduction

Microorganisms form surface-attached and matrix-enclosed biofilms in numerous ecosystems (Flemming and Wuertz, 2019). A conspicuous and ubiquitous feature of biofilms is the differentiation into physical structures (*i.e.*, architectures) and the formation of spatial patterns and stratifications at various scales. Despite the importance of spatial organization for ecological systems in general (Rietkerk, 2004), relatively little is known on the formation of the higher-order structures in complex biofilms. This is unexpected given that biofilm architecture seems to be related to critical processes in many benthic ecosystems (Battin et al., 2016; Findlay and Battin, 2016).

The study of the diverse physical structures of biofilms has been a mainstay in biofilm research. Reported architectures include cell clusters separated by voids (De Beer et al., 1994, 1996; Stoodley et al., 2001, 2002), mushroom-like caps, streamers extending into the bulk liquid (Hall-Stoodley et al., 2004; Stoodley et al., 2002), ripples (Neu and Lawrence, 2006; Stoodley et al., 1999), and honeycomb-like patterns (Battin et al., 2003; Guilbaud et al., 2015; Thar and Kühl, 2005). Redox balancing, mechanical instabilities, reciprocal interactions between growth and competition for nutrients, localized cell death, and grazing by protists have been invoked as endogenous drivers of biofilm morphogenesis (Xavier et al., 2009; Yan et al., 2019; Dietrich et al., 2013; Weitere et al., 2018).

The bed shear stress imposed by fluid flow is an important exogenous driver of biofilm structural differentiation. It can trigger sloughing and deformation of biofilms (Drescher et al., 2016; Stewart, 2012; Stoodley et al., 2001), or the formation of patterns such as migratory ripples (Stoodley et al., 1999). While dense and compact biofilms are better protected from shear stress and related drag fluid (Sudarsan et al., 2016; Wang et al., 2018), tall and exposed clusters are more susceptible to sloughing or displacement (Dunsmore et al., 2002). Furthermore, the production of extracellular polymeric substances (EPSs) can be modulated in response to high shear, conferring enhanced mechanical stability (Battin et al., 2003; Simões et al., 2007; Wang et al., 2014). Fluid flow also affects the thickness of the diffusive boundary layer, the establishment of chemical gradients and thereby resource replenishment and biofilm growth (Battin et al., 2003; Hondzo and Wang, 2002; Park et al., 2011; Picioreanu et al., 2009; Stewart, 2012; Stewart and Franklin, 2008; Stoodley et al., 1998). Thus, the link between fluid flow and both biofilm growth and structure is complex, influenced by the balance between shear-induced biomass loss and enhanced mass transfer and growth rate (Park et al., 2011; Simões et al., 2007; Hondzo and Wang, 2002; Wang et al., 2014). At the same time, biofilm architecture can also modify the local flow patterns. In fact, mathematical models and experiments have shown that troughs between adjacent clusters alter the flow fields around the latter, allowing for fluid flow around the basal layers of the biofilm, thereby enhancing mass transport to these otherwise nutrient-limited areas (De Beer et al., 1994; Picioreanu et al., 2000). Therefore, like in macroscopic landscapes such as coral reefs or forests, the structure of biofilms mediates in the biogenic construction of its immediate environment (Flemming et al., 2016).

Our current understanding of biofilm morphogenesis and patterning largely rests on mathematical modeling. For instance, external mass transfer resistance imposed by a thick diffusive boundary layer may be counteracted by the formation of finger-like and rough biofilm architectures (e.g., Van Loosdrecht et al., 2002; Picioreanu et al., 1998). Here, models have shown that tall and exposed clusters grow faster than smaller clusters because they access more of the limiting resource (e.g., nutrients, oxygen) in the bulk fluid. Local detachment can also influence biofilm morphogenesis. The erosion of structures protruding into the fluid flow may result in biofilms with low roughness, while sloughing, as linked to nutrient limitation at the biofilm base or the erosion of cells from the biofilm surface, can lead to rough and clumped architectures (Chambless and Stewart, 2007; Hunt et al., 2004).

Such modeling efforts have influenced experimental studies on biofilm formation and morphogenesis that, however, remain often limited to mono-species bacterial biofilms growing on agar plates or in flow cells (e.g., Bjarnsholt et al., 2013; Rossy et al., 2019). While such experiments have greatly advanced our understanding on the small-scale architecture of biofilms and interactions within them, they may poorly reflect the impact of complex fluid flow on biofilm morphogenesis and its characteristic length scale as often encountered in natural biofilm habitats, such as streams. In fact, complex biofilms, whether in stream ecosystems (Battin et al., 2016) or the intestine (Cremer et al., 2016), are often characterized by spatial patterns that exceed the dimensions of a typical flow cell. Approaches that provide high spatial resolution beyond the multi-millimeter scale are thus required to appreciate the governing physical, biological and ecological processes from which architectures and higher-order patterns emerge (Battin et al., 2007; Dzubakova et al., 2018; Milferstedt et al., 2009; Wagner et al., 2010; Yan et al., 2019).

Here we applied an experimental and multi-scale approach to relate the structure and function of phototrophic biofilms to their hydraulic environment. We used an automated optical coherence tomography (OCT) system (Depetris et al., 2019) to quantify the topography of the biofilm as a digital elevation model (DEM) over a large rectangular surface (0.025 m × 0.4 m). This approach is analogous to the *in situ* 3D imaging of forests and coral reefs providing quantitative and detailed structural information to test hypotheses relating structure to function of these ecosystems (Calders et al., 2020). Biofilms grew in experimental flumes along an increasing gradient of flow velocity and shear stress. Our general hypothesis was that differing space-occupancy strategies arise from trade-offs between flow-induced shear stress and nutrient replenishment, thereby resulting in a patterned microbial landscape. Guided by numerical simulations of flow across idealized biofilm structures and oxygen concentration microprofiles, we further show that biofilm structures are able to modify their local environment.

## Results and discussion

### Hydraulics affects biofilm architecture and patterning

**Figure 1:**
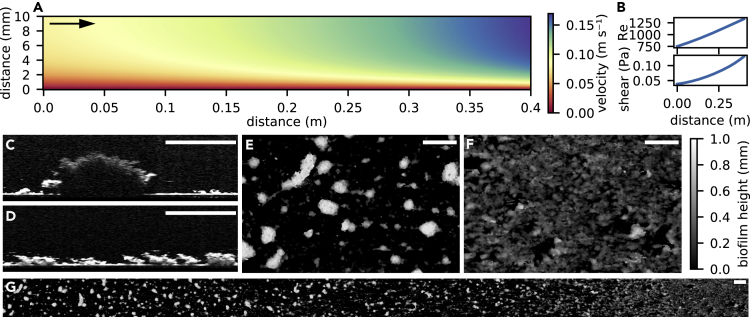
Architectural differentiation of phototrophic biofilms follows the hydraulic gradient The narrowing flume design from inlet to outlet (Figure S1) resulted in a gradual increase in flow velocity and bed shear stress in flow direction. An axial cross-section of the simulated velocity field is shown (A), with distance in the flow direction and from the flume bed on the *x* and *y* axes, respectively. Reynolds numbers and bed shear stress also increased in the flow direction (B) Optical coherence tomography (OCT) produced stacks of cross-sections at high resolution. Representative scans are shown for low (C) and high shear regions (D). OCT scans were processed to quantify the biofilm surface topology as a digital elevation models (DEM), in which biofilm thickness is encoded as pixel gray-level. Shown is a DEM covering a rectangle of 0.025 × 0.4 m^2^ along the hydraulic gradient (G), while enlarged details of the biofilm developed under low-shear (SFM) and high-shear (FFM) are also reported (E; F, respectively). Scale bars: 5 mm. Flow direction for all panels is indicated by the arrow in panel (A)

We cultivated phototrophic biofilms from raw water from Lake Geneva to mimic their growth in the lake outlet, the Rhone River. Biofilms grew over 15 days in duplicate open-channel flumes (I and II, 1.5 m long) with a geometry designed to produce a hydraulic gradient (Figures 1A and S1). Along this gradient, mean flow velocity ranged from 0.06 m s^−1^ to 0.13 m s^−1^, and Reynolds number for open channel flow (Supplemental information) increased from 793 to 1407, indicative of laminar to transitional flow, respectively. Numerical simulations further showed that the bed shear stress increased from 0.04 Pa to 0.13 Pa (Figure 1B). We used an automated OCT system (Depetris et al., 2019) to characterize the biofilm surface topology at high resolution (40 μm, 40 μm, and 2.18 μm in *x*, *y*, *z* dimensions) across several spatial scales — ranging from patches formed by multicellular clusters (~100 μm) to the higher-order patterns emerging at the scale of the entire biofilm landscape (0.4 m in length) (Figures 1C–1G). The OCT scans did not evidence the presence of voids below the biofilm surface and were processed to obtain DEMs of the biofilm surface topology (Depetris et al., 2019). After 15 days, biofilms developed into a clearly patterned landscape that followed the hydraulic gradient. Tall (up to 1.5 mm) clusters separated by troughs often containing small colonies (<100 μm in height) dominated the biofilm landscape exposed to low flow velocity and shear (Figures 1D and 1F). Biofilms growing under high flow velocity and shear stress developed into thin and coalescing patches densely carpeting the bottom of the flume (Figures 1E and 1G). The formation of these biofilm structures in complex phototrophic biofilms corroborates predictions from modeling and observed in other experimental systems (e.g., De Beer et al., 1994; Chambless and Stewart, 2007; Picioreanu et al., 1998; Stoodley et al., 2001, 2002). The contrasting morphotypes found at both extremes of the hydraulic gradient are hereafter referred to as SFM (slow-flow morphotype) and FFM (fast-flow morphotype).

**Figure 2:**
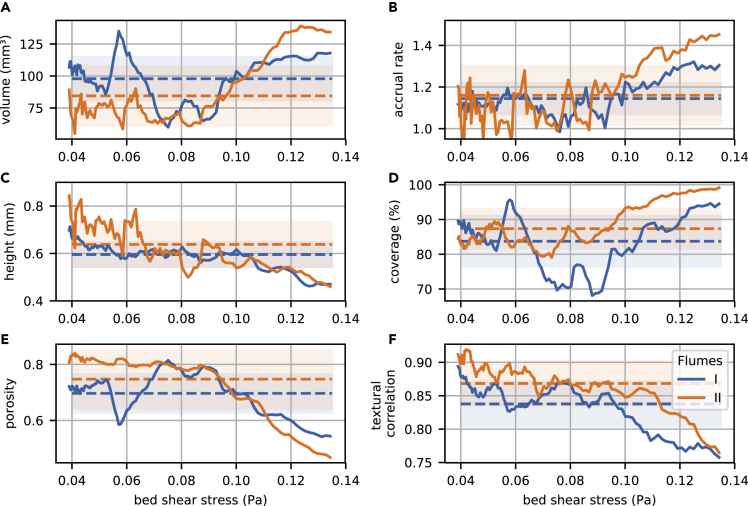
Key properties of phototrophic biofilms change along the hydraulic gradient Largescale OCT-derived DEMs were used to calculate moving window estimates (solid lines) of biofilm volume (A), accrual rate (B), height (or thickness) (C), coverage (D), porosity (E), and textural correlation (F) along the bed shear stress gradient (day 15; accrual rate was calculated between day 12 and day 15). Dashed lines and shaded areas depict overall averages and standard deviation, respectively. Gray bars indicate, when significant for both replicates, the range of changes in shear stress identified by breakpoint analysis (Table S2).

To quantify the changes in landscape features of mature biofilms (day 15) along the hydraulic gradient, we derived a suite of structural parameters from the DEMs using a moving window (24 mm edge length) approach (Table S1). While biofilm height gradually decreased with increasing shear stress, its volume, porosity, substrate coverage, and accrual rate exhibited significant breakpoints along the gradient in shear stress (Figure 2; Table S2). Biofilm volume, accrual rate, and substrate coverage presented a significant positive relationship with shear stress above the threshold of ~0.08 Pa but not below. This resulted in a more than 2-fold increase in biofilm accrual rate in FFM as compared to SFM. Shear-induced erosion can determine the height of biofilm structures (Chambless and Stewart, 2007), which would explain the observed gradual decrease in biofilm height with increasing shear stress. Given that biofilms developing under high shear are usually denser than those growing under low shear (Beyenal and Lewandowski, 2002), our results suggest that biomass accrual was more efficient under high shear. This is in line with previous experimental findings (Wang et al., 2014) and could be explained by enhanced mass transfer (e.g., Stoodley et al., 1998).

Above the breakpoint shear stress of ~0.08 Pa, biofilm porosity (*sensu* Picioreanu et al., 1998) and textural correlation, a measurement of biofilm aggregation (Haralick et al., 1973) tended to decrease with increasing shear stress (Figure 2, Table S2). Microorganisms compete, besides nutrients, and substrates, also for space, which can become a limiting resource for growth (Lloyd and Allen, 2015). Therefore, the presence of low coverage areas in SFM is remarkable and indicates the existence of processes limiting the full occupancy of the available space. Increased biofilm porosity and aggregation allowing for advection within biofilm troughs may be advantageous when mass transfer is limited under slow flow (De Beer et al., 1994; Picioreanu et al., 2000). Hence, the observed morphological pattern could represent an adaptive response to mass transfer limitation. Alternatively, clusters separated by voids could form as a consequence of detachment induced by nutrient limitation at the biofilm base combined with erosion of the cells on the biofilm surface (Chambless and Stewart, 2007). However, as noted by Stoodley et al. (2002), architectural differences may also underly differences in the community or EPS composition of the biofilm. Overall, these results suggest that the interplay between fluid flow and both biofilm architecture and growth dynamics are characterized by threshold effects as evoked by previous studies (Hondzo and Wang, 2002; Park et al., 2011; Simões et al., 2007; Wang et al., 2014), and further advocate contrasting mechanisms underlying biofilm morphogenesis.

### Limited community turnover between the SFM and the FFM

To test the hypothesis that contrasting morphotypes emerged from differences in community compositions, we sequenced amplicons of the 18S and 16S rRNA genes from the SFM and FFM (day 15) to identify eukaryotic and prokaryotic community members, respectively (Figure S3). Analysis of similarity (Anosim) based on Bray-Curtis similarities, showed no significant differences in community composition (16S rRNA: *R* = −0.15, p = 0.69; 18S rRNA: *R* = 0.11, p = 0.39) between the two morphotypes. The phototrophic community subset was dominated by Chlorophyceae, classified as *Scenedesmus* sp., contributing 36.6% to the reads in the 18S rRNA amplicon library, followed by *Mougeotia* sp. (Charophyta, 2.4%) and *Achanthidium* sp. (Diatomea, 2.3%). The bacterial communities were dominated by *Luteolibacter sp.* (Verrucomicrobiae, 4.7%), *Flavobacterium sp.* (Bacteroidetes, 2.7%) and not-further classified Sphingomonadaceae (Alpha-Proteobacteria, 2.5%) and Rhodobacteraceae (Alpha-Proteobacteria, 2.5%). *Scenedesmus* sp. is a small (~10 μm in diameter) coenobial and biofilm-forming microalgae that form structurally homogeneous biofilms under laminar flow (Zippel et al., 2007). However, in microbial communities developing under complex hydrodynamic conditions, microalgae may contribute to the formation of complex biofilm architectures (Lawrence and Neu 2003). Given its abundance, it is intuitive to assume that *Scenedesmus* sp. contributed to the here observed biofilm morphogenesis. Strikingly however, the same microalgal and bacterial communities were found in both SFM and FFM, reflecting their ability to form differing biofilm morphologies. Our findings evoke architectural plasticity, rather than contrasting community composition, as the process underlying morphological differentiation in the phototrophic biofilms here under study. Architectural plasticity is understood as the remodeling of the biofilms 3-dimensional structure as an adaptive response to their environment (Bridier et al., 2017). It involves, for instance, ecological interactions, the generation of physiological heterogeneities and the differential expression of the extracellular matrix components (Scheidweiler et al., 2019).

### Morphogenesis of the SFM and FFM

**Figure 3:**
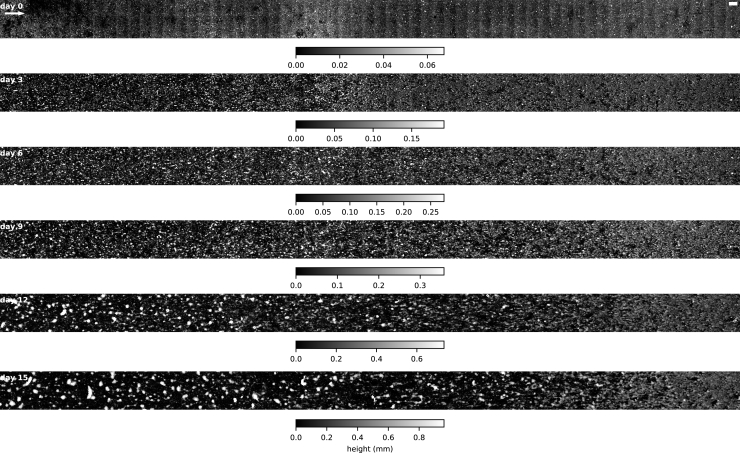
Biofilm morphogenesis over the course of the experiment The time series of biofilm morphogenesis for the entire hydraulic gradient in flume II is displayed. Note the differences in scales. The arrow indicates flow direction. Scale-bars: 5 mm.

**Figure 4:**
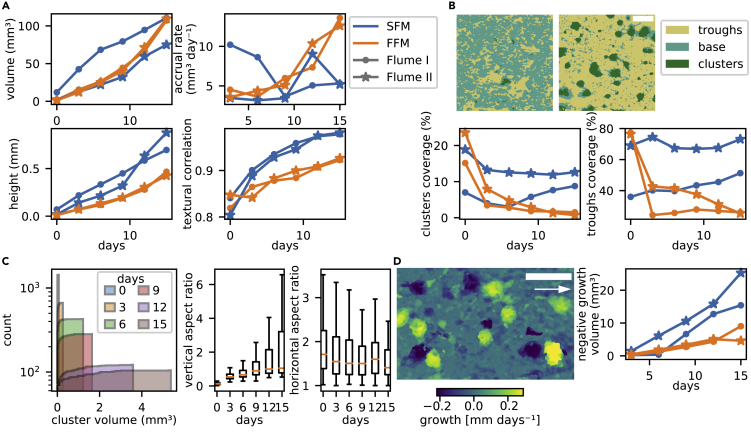
Biofilm morphogenesis differed across hydraulic conditions The temporal dynamics in biofilm volume, accrual rate, height and aggregation (textural correlation) for the SFM and FFM are shown in panel (A). A segmentation algorithm based on the difference from the local average thickness separated biofilm landscapes into base biofilm, protruding clusters and troughs (B). Shown are the temporal trends in the areal contribution of clusters and troughs. Cumulative histograms demonstrate the simultaneous increase in size and decrease in abundance of SFM clusters, while vertical and horizontal aspect ratios reveal their progressive elongation (C). Subtraction of subsequent DEMs (D, Figure S4) can be interpreted as a spatially explicit estimate of biofilm height variation. Areas with “negative growth” illustrated SFM clusters being detached from the substrate or displaced in flow direction (D). Total biofilm volume affected by “negative growth” increased over time and was higher in the SFM than in the FFM (D). The arrow indicates flow direction. Scale-bars: 5 mm.

DEMs of the biofilm surface topology acquired at 3-day intervals (Figure 3) allowed us to address biofilm morphogenesis dynamics. Volume accumulation curves did not differ between SFM and FFM but accrual rates steadily increased over time in the FFM and reached higher values than in the SFM (Figure 4A). Terminal accrual rates (day 12 to day 15) reached 9 mm^3^ day^−1^ and 14 mm^3^ day^−1^ in the FFM and 3 mm^3^ day^−1^ and 5 mm^3^ day^−1^ in the SFM (see also Table S1). The analysis of textural correlation showed that aggregation increased steeply at early time-points in the SFM, in contrast to the FFM, while biofilm maximum thickness was consistently higher in the SFM than the FFM at every time point (Figure 4A). Hence, the divergence into morphotypes started early during biofilm growth (day 3) and the SFM, despite being consistently thicker than the FFM, had lower accrual rates in later time-points. In agreement with previous reports (e.g., Hondzo and Wang, 2002; Park et al., 2011), elevated flow velocities, in the range tested here, had a net positive effect on growth, outweighing the effect of shear-induced erosion and scouring.

The presence of protruding biofilm clusters separated by empty areas can have important consequences for the local flow behavior, and related mass transport and drag force (De Beer et al., 1994; Picioreanu et al., 2000; Sudarsan et al., 2016). We compared the areal coverage of protruding clusters (*i.e.,* DEM areas at least two times larger than the local average) and the troughs (*i.e.,* DEM areas at least two times lower than the local average) between morphotypes (Figure 4B). The SFM was dominated by troughs (up to 73%) and clusters (up to 11%), whereas the FFM was largely devoid of such cluster-trough sequences and characterized by a base biofilm (up to 74%). The SFM clusters increased in volume (up to 4 mm^3^) but decreased in abundance over 15 days. They also exhibited an increasingly elongated aspect in the vertical direction and a slight elongation in the flow direction (Figure 4C). As mentioned above, cluster-trough sequences can enhance the external mass transfer toward the biofilm base (De Beer et al., 1994). At the same time, exposed clusters are more susceptible to the drag forces (Dunsmore et al., 2002; Sudarsan et al., 2016).

Despite the limited temporal resolution of the acquired OCT time-series, we observed that the biofilm structures that were dynamic over time. The dynamic rearrangement of the biofilm structures was particularly evident from the subtraction of subsequent DEMs (Figures 4D and S4), in which the local biovolume accumulation and displacement appear as positive and negative pixel gray-level values, respectively. At later time points, entire SFM clusters occasionally sloughed off, while others were apparently pushed in the flow direction. Overall, the FFM appeared more stable in time despite the higher shear stress. Roughly around the shear stress breakpoint, we observed the formation of structures reminiscent of migratory ripples (Figure S4; see also Stoodley et al., 1999). In the SFM, we found that up to 25 mm^3^ of biovolume was displaced between day 12 and 15, accounting for 33% of the total biovolume (Figure 4D). Hence, although we cannot rigorously quantify biomass detachment and displacement, these qualitative observations support the notion that taller biofilm structures are more exposed to drag force than more homogeneous and self-sheltering structures (e.g., Sudarsan et al., 2016; Dunsmore et al., 2002; Stoodley et al., 2002).

### Fluid flow within the SFM troughs provides solutes to the base of the biofilm

**Figure 5:**
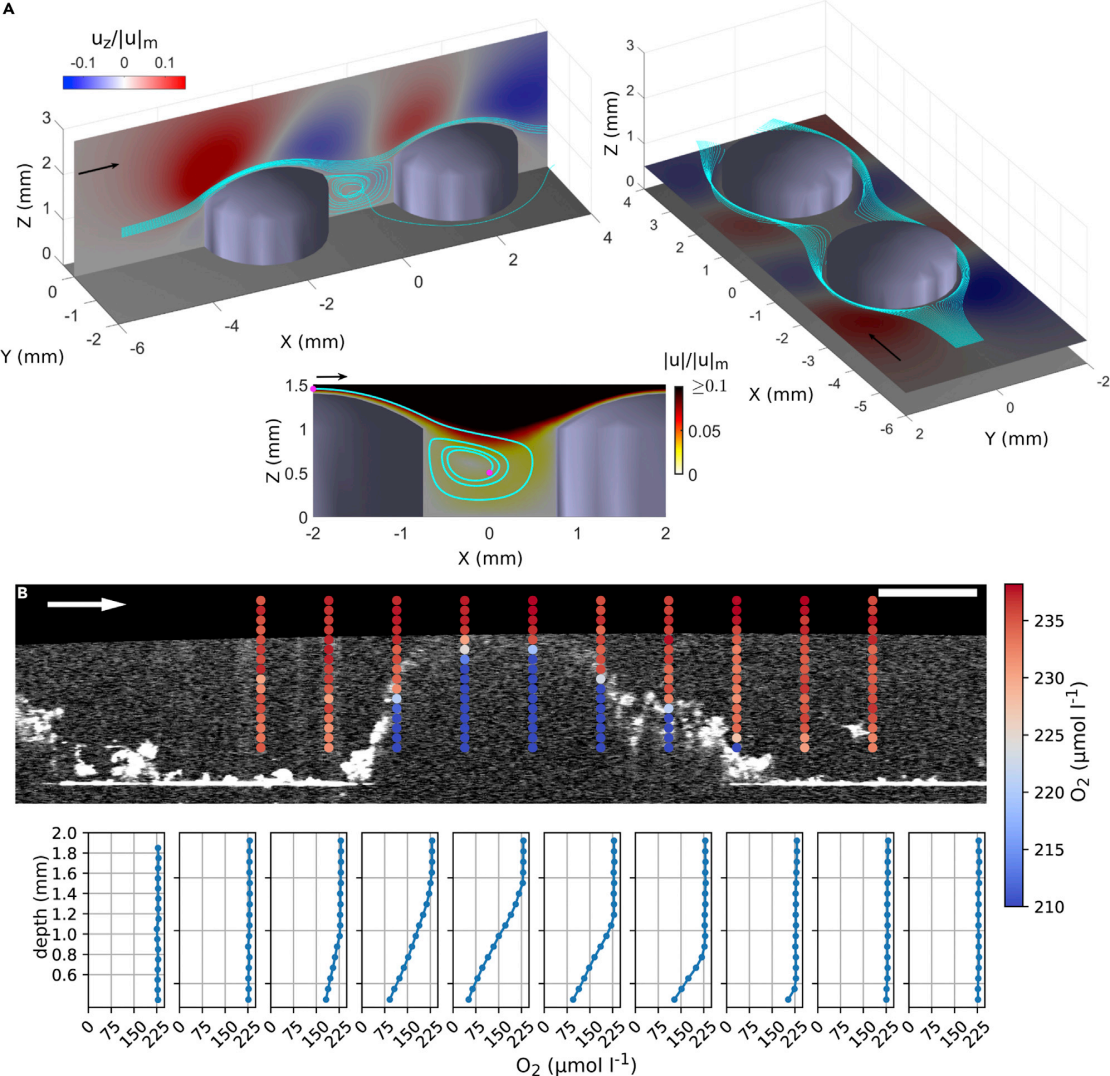
Numerical simulations and measured oxygen microprofiles suggest the presence of fluid flow around biofilm clusters Shown are the outcome of three-dimensional numerical simulations around two idealized biofilm clusters. (A) Provides the normalized vertical velocity and the lateral velocity components in the vertical symmetry plane (y = 0 mm) and a horizontal plane (z = 0.5 mm) superposed with selected streamlines. The lower subpanel shows the normalized velocity magnitude in the symmetry plane superposed with a streamline that demonstrates the fluid motion in the trough. (B) Shows representative oxygen measurements within and around a biofilm cluster (SFM) superimposed onto the respective OCT scan. Panel B illustrates oxygen microprofiles measured around and within biofilm clusters. Each point depicts the concentration of a single oxygen concentration measurement. Corresponding oxygen microprofiles are shown below. The arrow indicates the flow direction. Scale bar: 5 mm.

In order to assay the effects of surface topography on the local flow patterns, we used 3D numerical simulations to visualize the flow field around two idealized biofilm clusters with a width:height ratio similar to the SFM clusters. The simulated flow field revealed liquid flow into and within troughs between neighboring clusters and showed that fluid is advected into troughs predominantly via the vertical (*u*_z_) and the lateral (*u*_y_) components of velocity (Figure 5A). The simulated velocity field around two idealized biofilm clusters displayed downward and lateral fluid motions within the trough region between the clusters. Streamlines originating from upstream of the clusters entered the trough with velocities up to 10% of the mean flow velocity. Simulations further indicated that a fluid element entering the trough from the top recirculated in the trough region and reached the center of the region in approximately 1.5 min. Furthermore, we noted the formation of a vortex ring that could lead to enhanced fluid mixing in the trough region (Figure 5A). In line with previous models (Picioreanu et al., 2000), these simulations suggest that advection within troughs is relevant for solute transport and replenishment, which may be further facilitated by the formation of vortices.

Motivated by these simulations, we tested for the advective replenishment of oxygen within the biofilm troughs by measuring oxygen microprofiles across and around biofilm clusters (in darkness). Microbial respiration is expected to reduce oxygen concentrations in stagnant zones where diffusion is the sole source of oxygen replenishment (De Beer et al., 1994). However, we found that dissolved oxygen concentration did not differ between the troughs and the bulk fluid (Figure 5B), supporting the notion of oxygen replenishment by multi-directional advective transport. We did not detect a diffusive boundary layer around the biofilm structures in both the SFM and the FFM (sensitivity ~50 μm; Figures 5B and S5). These results agree with other empirical observations and modeling predictions from earlier studies on heterogeneous biofilms (De Beer et al., 1994; Picioreanu et al., 2000; Stoodley et al., 1997). However, the observation that the diffusive boundary layer around SFM clusters and troughs must be thin (<50 μm) eliminates external mass transfer resistance as a driver of the formation of a rough, finger-like architecture (Picioreanu et al., 1998). In contrast, both the measured oxygen microprofiles and fluid dynamic simulation suggest that the transport of solutes from the troughs toward the clusters may sustain microbial activity and growth at the biofilm base.

### Oxygen concentration profiles within biofilm architectures reveal chemical micro-niches

**Figure 6:**
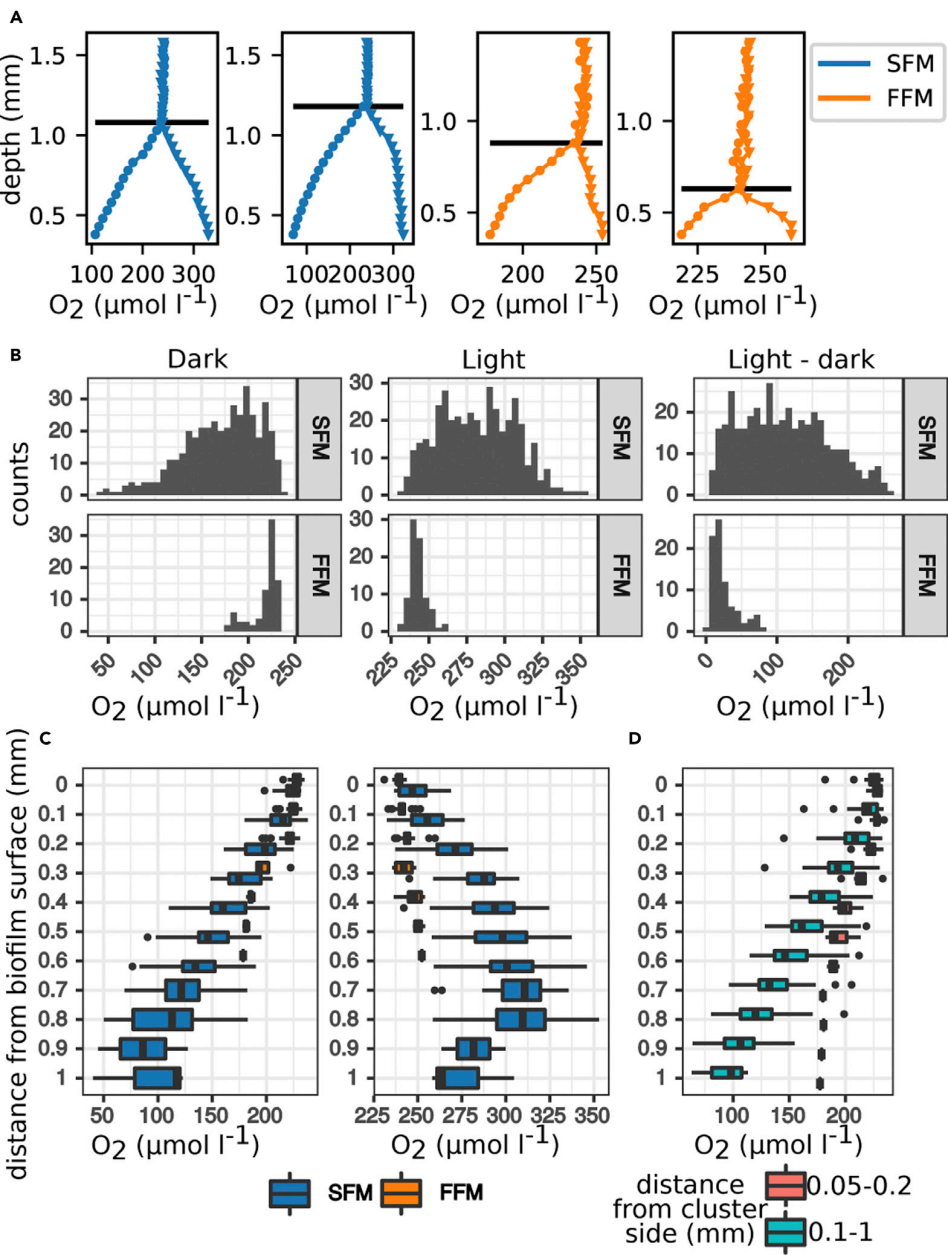
Oxygen microprofiling suggests a more heterogeneous distribution of chemical micro-niches in the SFM compared to the FFM Reported are representative oxygen microprofiles measured within SFM and FFM in light (triangles) and dark (circles) (A). Horizontal lines indicate the estimated position of the biofilm surface. Oxygen concentration and the difference in oxygen concentration between light and dark conditions were more heterogeneously distributed in the SFM than in the FFM (B). The distribution of oxygen concentration measured at the same biofilm depth (pooled in 100 μm steps) are compared between morphotypes (C) and at different distances from the SFM clusters walls (D, dark conditions).

Chemical gradients figure among the emergent properties in biofilms that result from internal mass transfer resistance and potentially drive the small-scale diversification of biological processes with large-scale consequences (Flemming and Wingender, 2010; Stewart and Franklin, 2008). For instance, limited mass transport within densely packed cells and their extracellular matrix can lead to nutrients deprivation in the deeper biofilm layers, potentially triggering sloughing (Chambless and Stewart, 2007; Hunt et al., 2004; Xavier et al., 2005). To map the spatial distribution of oxygen inside the biofilm, we measured multiple oxygen profiles within the SFM (n = 35) and FFM (n = 27) structures (few representative profiles are reported in Figure 6A). Oxygen concentrations (in light, dark, and the difference between light and dark) were more widely distributed in the SFM than in the FFM (robust Brown-Forsythe Levene-type test, p value < 0.01) (Figures 6B and Table S3). This corroborates the notion that structural heterogeneity, which is higher in SFM than FFM, leads to a heterogeneous distribution of chemical micro-niches in phototrophic biofilms.

Under dark conditions, dissolved oxygen reached a lower concentration in the SFM (40 μmol L^−1^) than in the FFM (178 μmol L^−1^), while the opposite was the case under light (SFM: 353 μmol L^−1^; FFM: 260 μmol L^−1^). Also, when comparing measurements at the same biofilm depths, oxygen concentrations reached more extreme values in the SFM compared to the FFM (t-tests, p *values* < 0.01 for both light conditions and depths into the biofilm from 0.05 mm to 0.25 mm) (Figure 6C and Table S4). These patterns are unlikely driven by differences in oxygen diffusivity, as biofilms grown under high shear are usually denser and less permeable to oxygen than their low-shear counterparts (Beyenal and Lewandowski, 2002). Hence, the gentler oxygen gradients within the FFM could be driven either by lower metabolic rates or advective oxygen transport inside the biofilm, but these processes cannot be disentangled in our experimental setup.

Next, we mapped oxygen concentrations across individual SFM clusters in dark conditions (Figure S6). We found that oxygen within SFM clusters was less depleted close to the cluster side walls compared to their inner cores (t-tests, p *values* < 0.01 for depths ranging from 0.1 mm to 0.5 mm) (Figure 6D and Table S5). This observation supports the notion that advective transport within troughs replenishes oxygen through SFM cluster walls. However, compared to the FFM, this effect only insufficiently supplies oxygen to the cores of SFM clusters, which were, compared to the FFM, more oxygen depleted in dark conditions (and more saturated when exposed to light). Given the low half-saturation coefficient for dissolved oxygen for bacteria (Hao et al., 1983), it is unlikely that oxygen itself limited the metabolic activity of the biofilms under study. However, it is conceivable that other solutes, such as nutrients and metabolic waste products, will follow similar spatial dynamics and potentially induce stress responses in the cores and at the base of the SFM. Therefore, differential detachment induced by nutrients limitation remains a potential mechanism that could underly the formation of clusters separated by voids under low shear.

We did not attempt to infer metabolic rates from the oxygen profiles because these could be compromised by advective transport within the biofilm matrix. However, we calculated the ratio between the oxygen gradients (from 0.05 to 0.15 mm depth) in light and dark as a proxy for net autotrophic *versus* heterotrophic aerial oxygen fluxes. Light:dark ratios averaged around 1 and were not significantly different between the SFM and the FFM (Welch's test, p *value* = 0.23, Figure S7), which indicates a balance between net oxygen production and consumption in both morphotypes.

### Conclusions

Combining 3D imaging with sequencing, numerical simulations of fluid flow, and oxygen microprofiling, we described architectural patterns in a complex phototrophic biofilm growing under a quasi-natural flow conditions. Over a relatively short hydraulic gradient, we observed strikingly diverging biofilm architectures despite limited community turnover. Clusters separated by troughs developed under low shear stress, while a more homogeneous biofilm layer covered the substrate under high-shear. Streams are characterized by a remarkable heterogeneity in hydraulic conditions, both on short- and large-spatial range. Our results indicate that the architecture of benthic biofilms may plastically respond to the hydraulic conditions in their habitat, even in the absence of strong shifts in the community composition.

Several experimental and modeling studies have previously investigated biofilms architectures under controlled laboratory conditions, providing numerous mechanistic hypotheses regarding their emergence. On one side, biofilm morphologies analogous to those observed here have been described across biofilm communities and spatial scales, and hence seem to be rather universal. On the other, our results confirm that the advective replenishment of oxygen within the biofilm troughs as well as the dynamic displacement and detachment of isolated clusters, may influence the morphogenesis of complex phototrophic biofilms in their natural environment. Our observations seem to exclude external mass transfer limitation as a driver of biofilm morphogenesis under the studied hydraulic conditions, whereas a combination of differential detachment induced by nutrients limitation and erosion of cells from the biofilm surface remains a viable explanation. We further observed an abrupt morphological transition around a threshold shear stress of ~0.08 Pa. Mechanical properties of the different architectures may lead to this threshold effect, however this remains to be addressed in future theoretical and empirical studies. Overall, our findings suggest that a tight coupling between architectural plasticity, chemical heterogeneity and mechanical resistance to shear affects the formation of the meso-scale patterns observed in biofilms that coat the benthic zones in streams and rivers.

### Limitations of the study

Following an approach often used in landscape ecology (Calders et al., 2020), we inferred the functional links between hydraulic conditions and biofilm morphogenesis from their spatial correlation. While this approach allowed us to study complex phototrophic communities at high resolution and under realistic hydraulic conditions, we cannot account for all potential effects. For instance, biomass may accumulate downstream, potentially oozing in the direction of the flow. This possibility seems unlikely, as shear and turbulence in the flume fast-flow region were strong enough to detach biomass that was just loosely adherent to the substrate, and did not permit the sedimentation of aggregates from the water column (visual observation). Furthermore, we started detecting a morphological shift between contrasting hydraulic conditions at early stages in biofilm development (3 days), when the accumulated biovolume was still remarkably low and no significant displacement of the biomass occurred yet. Nonetheless, the relationship between biofilm architecture and hydraulic conditions remains correlational and further experiments would be required to rigorously test any causal link. Working with complex communities and natural surface water as growth medium allows the formation of diverse biofilms relevant for stream ecosystems, but seasonal variation in inflow medium and seed community reduced the repeatability of the experiments. However, the communities that we sampled and the patterns that we observed are realistic and informative. The raw lake water used was filtered (nominal pore size 50 μm), but this removed just larger grazers and did not completely eliminate grazing pressure, which could have further modulated the morphology of the studied biofilms (Böhme et al., 2009; Weitere et al., 2018). The flumes were constructed from plexiglass and this smooth substrate may have affected the observed dynamics to some extent. Nonetheless, the two biofilm morphotypes exhibited structural differences leading to diverging structural resistance, which are likely independent on the type of substrate colonized. OCT has a limited penetration depth within biofilms and imaging depth in water; as a consequence, structures taller than ~1.2 mm were not reliably imaged, which may have caused an underestimation of the total volume, accrual rate and biofilm thickness, particularly at later time-points and under slow flow.

### Resource availability

#### Lead contact

Further information and requests for resources should be directed to and will be fulfilled by the Lead Contact, Tom Battin (tom.battin@epfl.ch).

#### Materials availability

This study did not generate new unique reagents.

#### Data and code availability

Raw OCT scans, processed DEMs, and oxygen microprofile data have been deposited at https://figshare.com/projects/Data_for_Morphogenesis_of_phototrophic_biofilms_is_controlled_by_hydraulic_constraints_and_enabled_by_architectural_plasticity/87296. Raw sequences are available at the European Nucleotide Archive (ENA) under accession number PRJEB39886. Code used for image acquisition, processing and analysis is available under https://figshare.com/projects/Data_for_Morphogenesis_of_phototrophic_biofilms_is_controlled_by_hydraulic_constraints_and_enabled_by_architectural_plasticity/87296.

## Methods

All methods can be found in the accompanying Transparent Methods supplemental file.

## Supplemental information

Document S1. Transparent methods, Figures S1–S7, and Tables S1–S5

## Footnotes

Supplemental information can be found online at https://doi.org/10.1016/j.isci.2021.102067.
