# Supplementary material for: Morphogenesis and oxygen dynamics in phototrophic biofilms growing across a gradient of hydraulic conditions
Source: iScience. 2021 Jan 20;24(2):102067. doi: 10.1016/j.isci.2021.102067 (PMC7868926; doi:10.1016/j.isci.2021.102067)
Supplement: Document S1. Transparent methods, Figures S1–S7, and Tables S1–S5 [file mmc1.pdf]

iScience, Volume *24*

Supplemental Information

Morphogenesis and oxygen dynamics in phototrophic biofilms growing across a gradient of hydraulic conditions

Anna Depetris, Hannes Peter, Ankur Deep Bordoloi, Hippolyte Bernard, Amin Niayifar, Michael Kühl, Pietro de Anna, and Tom Jan Battin

Supplemental Tables

Table S1. Morphological features of SFM and FFM at the two extremes of the flow velocity gradient after 15 days of growth, Related to Figure 1. The reported accrual rates refer to the time interval between day 12 and day 15.

		**hydraulic conditions**		**morphological features**					
	flume	flow velocity [m s^{-1}]	shear stress [Pa]	volume [mm^3]	accrual rate [mm^3 day^{-1}]	height [mm]	coverage [%]	porosity	textural correlation
SFM	I	0.059	0.04	107	3.73	0.7	90	0.72	0.89
	II	0.059	0.04	89	5.04	0.84	85	0.81	0.91
FFM	I	0.135	0.13	118	9.18	0.47	95	0.54	0.76
	II	0.135	0.13	134	13.96	0.46	99	0.47	0.76

Table S2. Segmented-regression analysis of the morphological gradient, Related to Figure 2. We tested for the presence of a breakpoint, separating two regions of the morphological gradient with contrasting relationship with shear stress, using the Davies' test for a non-zero difference-in-slope parameter of the segmented regression. The bed-shear stress breakpoint, standard error (SE) estimates and p-vlaues are reported as well as the adjusted R^2 of the segmented regression. When the Davies' test was not significant, the adjusted R^2 of a linear model is reported. The statistical significance of trends below and above the breakpoint shear stress (low and high shear respectively), was tested with the modified version of the Mann-Kendall test for autocorrelated series. Significant trends were evaluated with a linear model, whose slope are reported (units of the variable * Pa^{-1}). When no significant breakpoint was observed, the Mann-Kendall test was performed on the entire data set.

		**Davies' test**				**Mann-Kendall test**				**linear model**
						SFM		FFM		
	flume	breakpoint shear [Pa]	SE	p-value	adj. R^2	corrected Z	p-value	corrected Z	p-value	slope
volume [mm^3]	I	0.085	0.003	<0.05	0.50	-1.85	0.06	2.63	<0.01	1108
	II	0.084	0.002	<0.01	0.92	-0.62	0.53	3	<0.01	1661
accrual rate [mm^3 day^{-1}]	I	0.089	0.006	<0.01	0.78	-1.16	0.24	2.63	<0.01	190.5
	II	0.083	0.006	<0.05	0.89	-0.62	0.53	3.00	<0.01	281.9
height [mm]	I	-	-	0.19	0.78	-3.3	<0.01	-	-	-1.69
	II	-	-	0.26	0.76	-4.2	<0.01	-	-	-3.02
coverage [%]	I	0.087	0.002	<0.01	0.67	-2.26	0.02	2.63	<0.01	568
	II	0.073	0.002	<0.01	0.86	-0.72	0.47	3.34	<0.01	314
porosity	I	0.089	0.002	<0.01	0.68	1.44	0.15	-2.63	<0.01	-5.7
	II	0.089	0.001	<0.01	0.99	-2.81	<0.01	-2.63	<0.01	-7.5
textural correlation	I	-	-	0.10	0.75	-2.98	<0.01	-	-	-1.07
	II	0.111	0.002	<0.01	0.92	-12.83	<0.01	-1.04	0.3	-0.76

Table S3. Statistics of the distribution of the dissolved oxygen concentration in the slow- and fast- flow morphotypes (SFM and FFM), Related to Figure 5 and Figure 6. We obtained several profiles of dissolved oxygen concentrations in different locations within the SFM and FFM, under light and dark conditions (flume II). The features of the distribution of oxygen concentrations (points from all profiles were pooled) are reported.

		**FFM**	**SFM**
	number of profiles	27	35
	number of points	84	406
dark [µmol O_2 L^{-1}]	0.1 quantile	189.9	115.9
	0.9 quantile	230.9	219.2
	maximum	235.1	238.1
	mean	218.1	170.8
	median	224.2	177.1
	minimum	177.9	40.2
light [µmol O_2 L^{-1}]	0.1 quantile	237.0	250.9
	0.9 quantile	250.7	313.6
	maximum	259.8	352.9
	mean	242.6	282.6
	median	241.8	282.8
	minimum	230.9	232.5

Table S4. Test statistics, Related to Figure 6. T-tests for unpaired samples with uneven variances to test for differences in oxygen concentration at different depth within the biofilm among profiles taken within the SFM and FFM in light and darkness, respectively. Measurements were pooled in 0.1 mm steps.

depth within biofilm [mm]	dark p-value	light p-value	n_{SFM}	n_{FFM}
0.05	2.7e-02	1.17e-04	24	24
0.05-0.15	1.12e-06	4.40e-11	58	24
0.15-0.25	4.57e-13	6.73e-22	86	23
0.25-0.35	4.74e-02	6.16e-06	32	4
0.35-0.45	2.73e-10	3.19e-06	74	5
0.45-0.55	2.38e-08	2.84e-10	39	3

Table S5. Test statistics, Related to Figure 6. T-test for unpaired samples with uneven variances to test for differences in oxygen concentration at different depths within the biofilm among profiles taken within the SFM clusters in the dark. Measurements were pooled in 0.1 mm steps.

depth within biofilm [mm]	p-value	$n_{dist[0.05-0.2]}$	$n_{dist[0.2-1]}$
0.05	0.22	6	14
0.05-0.15	0.03	9	33
0.15-0.25	0.003	9	33
0.25-0.35	0.004	7	32
0.35-0.45	0.008	6	30
0.45-0.55	0.004	5	29

Supplemental Figures

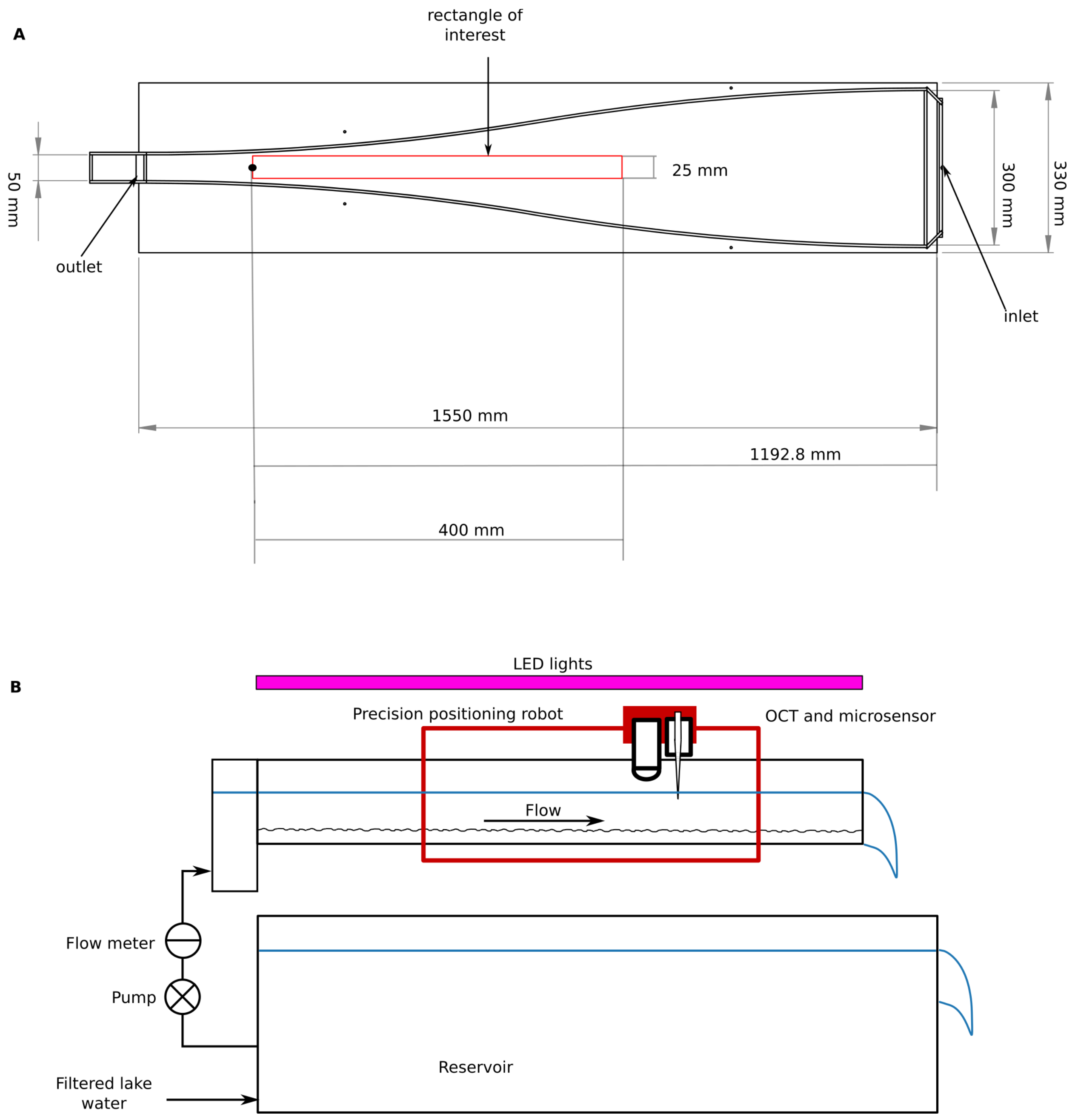

Supplemental Figure S1. Experimental Design, Related to Figure 1. Plexiglass flumes were designed with the represented geometry to obtain a flow velocity gradient, increasing in the direction of the flow. The "rectangle of interest" indicates the position of the biofilm characterized in this study. The flumes had a total depth of 50 mm, while the depth of the water was ~ 22 mm (A). Filtered lake water was continuously pumped into a large reservoir, from which it was pumped into the flume. The flow rate was controlled with a flow meter. The water was fully recirculated into the reservoir. Light was provided with LED lights, while the OCT probe and the microsensor were mounted on a robot that allowed to precisely control their positioning (B).

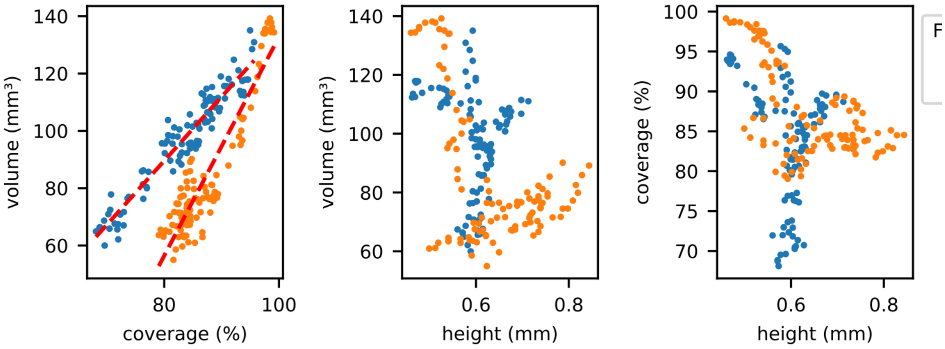

Supplemental Figure S2. Biovolume is correlated to surface coverage rather than to biofilm height, Related to Figure 2. We found a significant positive correlation between the volume accumulated in a square moving window (24 mm edge length) along the hydraulic gradient (flume II is showed in Figure 1 of the main text) and total coverage in the same window (lm, $slope_I = 2.23$; $slope_{II} = 3.82$; $R^2_I = 0.96$, $R^2_{II} = 0.93$; p-value < 0.01 for both). In contrast, maximum height of the biofilm, calculated as the 0.99 percentile of the height distribution, was found to have a negative and non-linear relationship with accumulated volume.

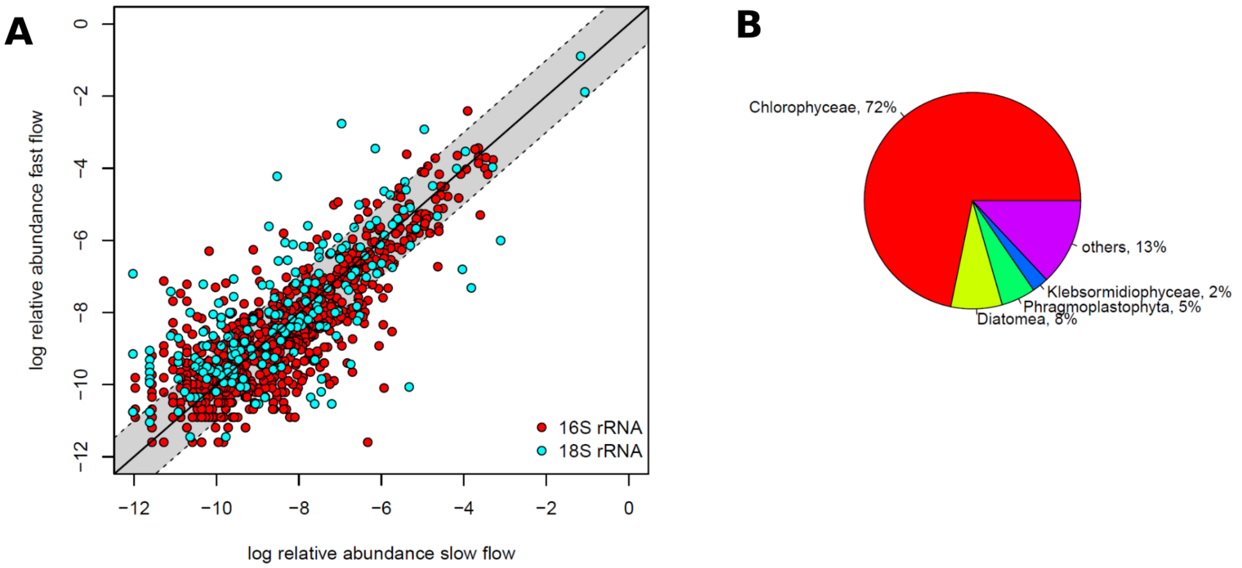

Supplemental Figure S3. Community composition in experimental biofilms, Related to Figure 1. Community composition did not differ between SFM and FFM morphotypes (A). Shown are average relative abundance (log) for 16S rRNA and 18S rRNA sequence variants for biofilms sampled from slow and fast flow areas. Only sequence variants that occurred in both morphotypes are shown (n_{16S} = 779, cumulative relative abundance: 96.9% of the entire 16S rRNA dataset; n_{18S} = 390, cumulative relative abundance of the entire 18S rRNA dataset: 98.3%). The 1:1 line (solid line) and the grey shaded area denoting ± 1 log units from the 1:1 line are shown for guidance. (B) Visual and microscopic inspection showed that biofilm biomass was dominated by phototrophic microorganisms. Refining the 18S rRNA gene dataset to include taxonomically resolved Archaeplastida, Stramopiles, Alveolates and Rhizaria (the three latter being combined to the supergroup SAR), we retained 173 sequence variants. Together, they represented 53.3% of relative read counts for the 18S rRNA dataset, with Ophistokonta and Amoebozoa representing another 44.8%. The phototrophic communities included 18 taxonomically resolved classes, which were dominated by Chlorophyceae (71.7% relative read counts), Diatomea (7.7%), and Zygnematophyceae (5.1%). Chlorophyceae included 13 distinct 18S rRNA sequence variants, however, across all samples, they were dominated by a single ASV (contributing to 95% of reads classified as Chlorophyceae). A blast search of this sequence resulted in hits to Scencedesmus sp. (blastn against nr/nt database, max score 695, E-value < 1e-179). Scenedesmus is a common, colony-forming (4 - 30 elongated cells laterally joined), non-motile and high-ligth adapted freshwater green algae (Chlorococcales), often found in stream biofilms (e.g. Pohlon et al., 2010; Sherwood, 2016), and can pioneer biofilm succession (Roeselers et al., 2007). The phototrophic biofilms were further composed of diverse Diatomea predominantely classified as Bacillariophytina (with 12 sequence variants and 5.2 out of 7.7% overall contribution of Diatomea to phototrophic community read counts) and Fragilariales (6 sequence variants and 2.4% out of 7.7% contribution). Both Bacillariophytina and Fragilariales are common in stream and river biofilms, where they often dominate carbon fixation (Burliga and Kociolek, 2016). Commonly described as diatoms, these taxa often assume a benthic livestyle in flowing waters, facilitated by fibrillar attachment structures (Wang et al., 2014) and the production of extracellular polymeric substances (EPS) which are released from the apical field of pennate diatoms. Mougeotia sp. (Phragmoplastophyta, Zygnematophyceae) is a cosmopolitan mat-forming freshwater algae and may occur in later stages of biofilm formation (Stelzer and Lamberti, 2001). Taken together, our experimental biofilms were composed of a diverse assemblage of common freshwater biofilm forming taxa, such as found in many streams and rivers.

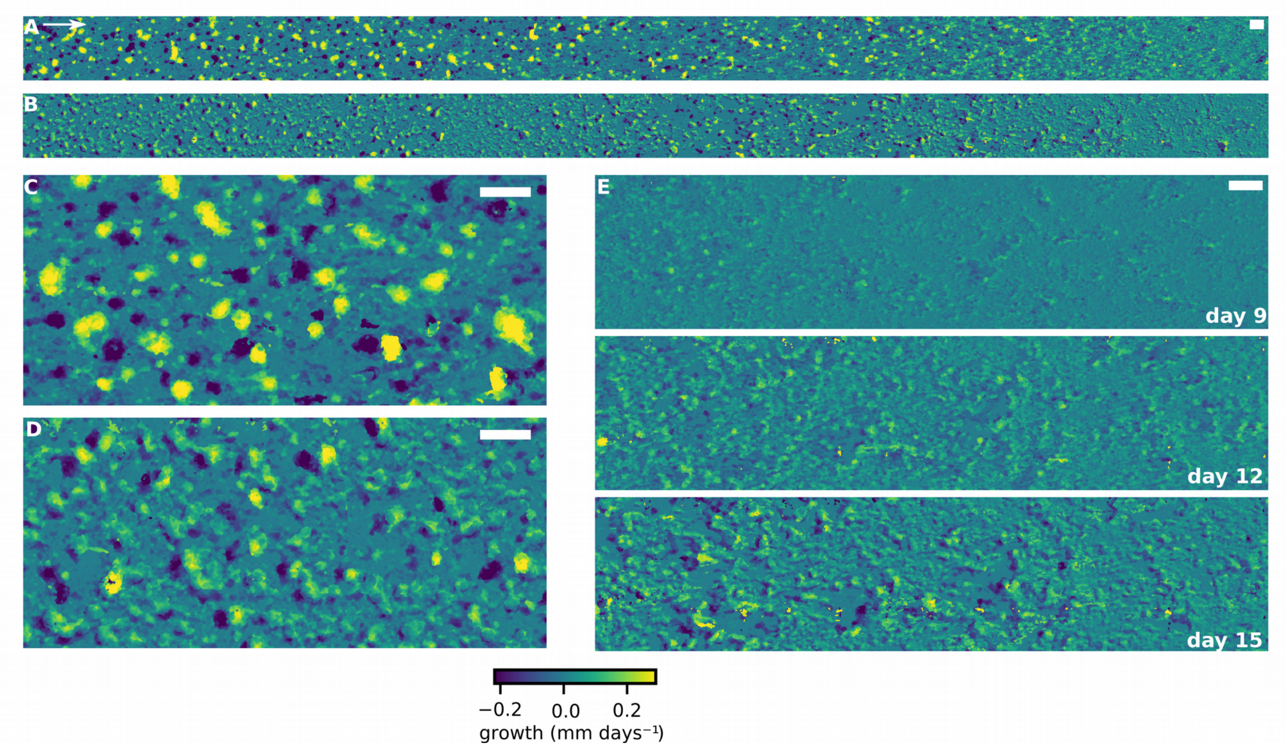

Supplemental Figure S4. Estimating displacement, Related to Figure 4. The OCT derived DEMs obtained at subsequent time points were subtracted to obtain the rate of increase in biofilm height per day. Panels A and C refer to flume II between day 12 and 15, while B and D to flume I between the same time-points. We attribute areas with negative accrual rates to displacement of biofilm clusters. Panel E shows the progressive enlargement of empty areas in the flow direction and the formation of structures resembling migratory ripples at the transition between the two morphotypes (time points are indicated). Scale-bars: 5 mm.

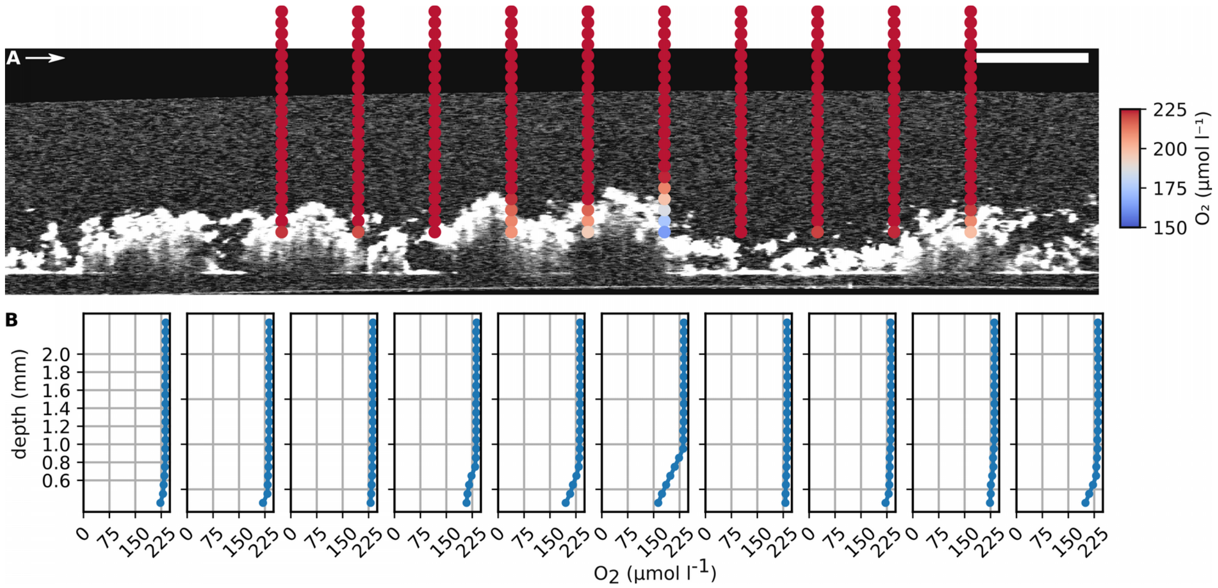

Supplemental Figure S5. Oxygen microprofiling, Related to Figure 5 and Figure 6. Shown are representative oxygen measurements within and around a biofilm structure (FFM) superimposed onto the respective OCT scan. Each point depicts the concentration of a single oxygen measurement in darkness (scale bar: 5 mm, arrow indicates the flow direction). The corresponding oxygen microprofiles are shown below. Note the absence of a diffusive boundary layer.

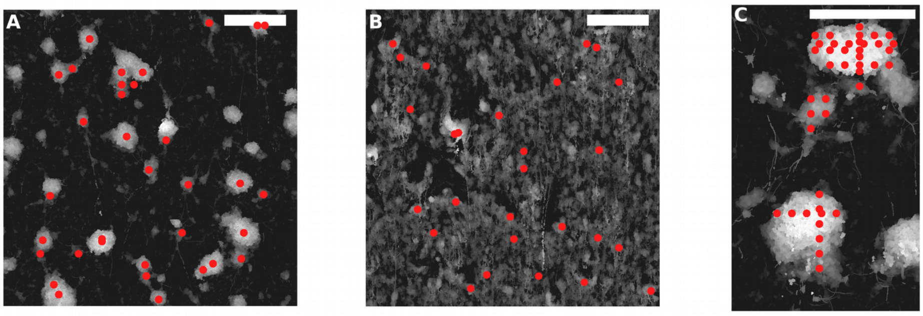

Supplemental Figure S6. Positions of the measured profiles, Related to Figure 6. To assess the spatial heterogeneity of the concentration of oxygen, we measured 35 and 27 oxygen profiles in both light and dark, for the slow flow morphotype (SFM) (A) and fast-flow morphotype (FFM) respectively (B). The locations of these profiles are indicated by the red dots. Further, we obtained 24 oxygen profiles in different positions within two clumps of the SFM, in dark conditions (C). Scale-bars are 5 mm.

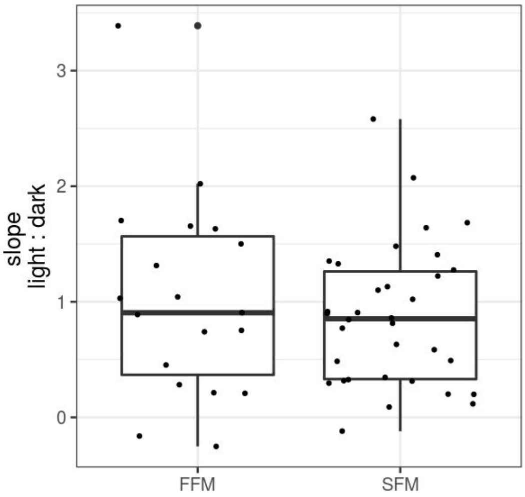

Supplemental Figure S7. Autotrophic and heterotrophic balance, Related to Figure 6. The ratio of the slope of the O_2 gradient below the biofilm surface (between 0.05 and 0.15 mm within the biofilm, light:dark) indicated a balance between heterotrophic and autotrophic aerial oxygen flux in SFM and FFM.

Transparent Methods

Flume experiments. Water from Lake Geneva was continuously filtered (nominal pore size 50 µm, FA 10 SX 50 ATLAS FILTRI) into a 1 m^3 reservoir and from there pumped into open-channel flumes. A constant flow rate (2.22 10^{-4} m^3 s^{-1}) was supplied using valves equipped with flow meters. Water was recirculated in a large reservoir, in which the water level was kept constant with a continuous inflow from the lake (8.3 10^{-6} m^3 s^{-1}). The total water volume in the flume was ~ 5.8 10^{-3} m^3, resulting in an average residence time of 26 s. Water temperature varied between 14.5°C and 15.5°C throughout the experiments, while pH oscillated between 7.16 and 7.55 (7.72 ± 0.30 [mean ± standard deviation]). Every 3 - 5 days, a water sample was filtered (0.22 µm polycarbonate filters, Isopore, Millipore) and analyzed by ion chromatography (Metrohm 930 Compact). Nitrate concentrations averaged 2.5 ± 0.4 mg l^{-1} and nitrite was below detection limit (< 0.05 mg l^{-1}). Further, total dissolved organic carbon (DOC) was quantified in filtered sample (pre-ashed GF/F, Whatman) using a TOC carbon analyzer (Sievers M9 TOC Analyser, GE). DOC varied between 872 and 3580 ppb, and averaged 1706 ± 731 ppb. Ammonia was quantified spectrophotometrically as described in (Taylor et al., 2007), and averaged 4.7 ± 0.9 µg l^{-1}. Soluble reactive phosphorous was quantified using the methylene blue method and averaged 2.0 ± 1.3 µg l^{-1}. Light (~13 W m^{-2}, JAZ spectrometer Ocean Optics) was provided for 12 hours per day using a combination of red and blue LEDs. The flumes were constructed from plexiglass with a funnel-like shape, imposing a gradient of flow velocity and bed shear stress on phototrophic biofilms. Flume width decreased gradually from 0.3 m to 0.05 m, with a slight curvature (**Supplementary Figure 1**). Average water depth was 0.022 m. The inflow was equipped with a round shaped adapter and a diffuser to minimize upstream turbulence. Prior to experiments, phototrophic biofilms growing continuously in the reservoir were harvested and disaggregated by shaking. The slurry was filtered using (41 µm nylon filter, Millipore) and diluted into 8 L of lake water. The biofilm slurry was then poured into the flumes and incubated for 12 hours without flow (under light). This seeding resulted in a thin layer of base biofilm evenly covering the flume bottom. After seeding, the flow was started and biofilm was allowed to grow without disturbance for 15 days.

Hydraulic gradient characterization. Mean flow velocity (u) and Reynolds number (R) were estimated from the flume geometry, the water depth (0.022 m), and discharge (2.22 10^{-4} m^3 s^{-1}) using formulas (1,2).

$$u=\frac{Q}{h \times w} \qquad (1)$$

$$R=\frac{Q}{v \times (2 \times h+w)} \qquad (2)$$

where *v* is the kinematic viscosity of water, *Q* is the discharge, *w* is the flume's width and *h* is the water depth.

The flow velocity within the open-channel flow chamber without biofilms was modeled using the geometrical design of the flume (Supplementary Figure 1). We used FLUENT (ANSYS R.19) to numerically solve a multiphase model (Volume of Fluid method), including 0.022 m water phase at the bottom of the flume, and a 0.018 m thick volume of air on top. We used the Reynolds-averaged Navier-Stokes approach, with a no slip-boundary condition on the plexiglass surface. An SST k-ω turbulence model (Menter, 1993), that is a two-equation eddy-viscosity model, was used to model turbulence. SST k-ω ensures simulation accuracy across the flow field by employing k-ω turbulence model in the regions near the wall and k-ε in other regions. The flow was generated by a pressure gradient between inlet and outlet. In order to resemble the flume flow field, boundary conditions were set similar to the flume operating conditions where the inlet discharge was 2.22 10^{-4} m^3 s^{-1}. The

results of the simulation were used to estimate the velocity magnitude in every point, calculated as the sum of the x- y- and z- vector components of velocity.

Bed shear stress was estimated as

$$\tau_s = \mu \left(\frac{du}{dy} \right)_{y=0}$$

where μ is the dynamic viscosity of water at 15°C.

OCT positioning and imaging. We used a spectral domain optical coherence tomograph (GANYMEDE, Thorlabs GmbH, Germany) centered at 930 nm and equipped with an immersion adapter. OCT datasets were acquired by averaging 3 A-scans and had a resolution of 40 µm in *x* and *y* and 2.18 µm in *z* direction, covering a volume of 10 mm × 10 mm × 2.23 mm. We mounted the OCT probe on a precision positioning device (STEPCRAFT, 30 µm precision in x,y), as described previously (Depetris et al., 2019). Automatic positioning and OCT scan acquisition allowed us to obtain 66 × 3 OCT scans in a mosaic pattern (overlap was set to 30 % of the field of view). The final stitched OCT covered a total field of 0.040 × 0.0024 m. Further, higher resolution (11 µm *x, y* and 2.18 µm *z)* images in tiled patterns of 3 × 3 were taken at the two extremes of the velocity gradient. Biofilms were imaged every 3 days.

OCT scan processing and analysis. OCT scans were corrected for the lateral distortion using a custom made algorithm (Depetris et al., 2019). Then, OCT scans were binarized and the thickness of the biofilm at each position encoded in a digital elevation model (DEM). DEMs were cropped ~ 50 µm above the plexiglass surface; this represents the minimum height of structures considered in this work. Shorter structures were disregarded, in order to avoid fine irregularities of the plexiglass. Elevation maps were then stitched and denoised with a median filter. Biovolume was estimated as the volume beneath the biofilm surface, (*i.e.,* the sum of all pixels values in the DEM). This was supported by visual inspection of the OCT images, which did not reveal the presence of voids below the biofilm surface. Biofilm accrual rate over 3 days was calculated as the difference in biovolume between subsequent time-points, divided by the time lag (3 days). Biofilm maximum thickness was calculated as the 0.95 percentile of the height distribution; coverage was calculated as the percentage of the pixels within a DEM with values greater than zero. Biofilm solids hold-up was calculated as described by Picioreanu et al. (Picioreanu et al., 1998), as the ratio between biofilm volume and the volume of a parallelepiped with a basal area equal to the substrate's surface area and height equal to the maximum biofilm height. Porosity was them calculated as 1 - biofilm solids hold-up. Textural correlation was calculated from the gray-level co-occurrence matrix (Haralick et al., 1973) using the *scikit-image* package GLCM Texture Features, with distance = 200 µm and angle = 0°. Setting the distance parameter from 0 to 4000 µm and angles 0°, 90°, 180° did not qualitatively change the results. A square window of 0.024 m edge was moved along the stitched DEM in steps of 4 mm. For the fragmented regression analysis, we applied the Davies test to test for the presence of a breakpoint (function *davies.test* in R). Then, the position of the breakpoint and the slopes of the two contrasting trends were estimated using the '*segmented'* R package using 0.11 Pa as a starting value (Muggeo, 2008). The significance of the trend in biofilm architectural features with increasing shear was tested within the two fragments (or on the entire dataset when the Davies test did not evidence a significant breakpoint) using a modified version of the Mann Kendall test for autocorrelated data (Hamed and Ramachandra Rao, 1998), using the R function *mmkh* (from the package '*modifiedmk*'). Finally, in the absence of a significant breakpoint, the slope of the significant trends was estimated with a linear model. For these analyses, the dataset was reduced to include only non-overlapping windows and avoid excessive spatial autocorrelation. Linear regressions for the relationship between volume, height and coverage were also computed using R (R Core Team, 2020). Image processing and analyses were done using Python, NumPy, scikit-image package when not stated otherwise. Jupyter notebook are available here. To analyze biofilm morphogenesis, we utilized higher resolution OCT scans (11.1 µm, 11.1 µm, 2.18 µm in *x, y, z*),

which were processed analogously to the lower resolution counterparts. Volume, height, coverage and textural correlation were calculated as before. To segment troughs, clusters and valleys, we applied a gaussian filter with sigma 2.2 mm and selected areas that were 2 times taller than the filtered image as clusters, and 0.5 times lower as troughs. Segmentation parameters were chosen on the basis of a visual checks. The shape of individual clusters was analyzed using the *regionprops* function of the *scikit-image* package. Volume was calculated as the sum of pixels in the portion of the DEM containing the cluster of interest. The horizontal aspect ratio was calculated as the ratio between the major and minor axes of the ellipse that has the same normalized second central moment as the region basal area. Orientation represents the angle of the major axis with respect to the flow direction. The vertical aspect ratio was calculated as the ratio between cluster height and the square root of its basal area. Displaced volume and coverage were calculated by subtracting subsequent DEMs in the time series and selecting areas with negative values.

Fluid dynamics simulation around two idealized biofilm clusters. We simulate the 3-dimensional laminar stationary flow around two idealized biofilm clusters using Comsol Multiphysics 5.4. The computational domain represents a sub-region from our experiment, and it consists of a rectangular block with dimensions L_x = 16.5 mm, L_y = 7.5 mm and L_z = 4.5 mm. A right-handed co-ordinate system is centered at the center of the bottom surface. The idealized bio-film clusters are modeled as two impermeable rigid vertical structures each comprising of a circular cylinder (radius = 1.25 mm, height = 1 mm) capped with a truncated hemisphere of radius 2.15 mm. The two cylinders are symmetrically located on the bottom surface along the streamwise (x-axis) direction with center-center distance of 4 mm. We provide an inlet flow condition of uniform mass flux of 0.2 g s^{-1} chosen strategically based on experimental conditions. The boundary conditions are applied such that each boundary represents that of a sub-volume in the experimental field; no-slip on the bottom surface including the surface of each cluster, open-flow condition (with zero viscous stress) on the top surface, and periodic boundary conditions are applied on the two side surfaces. We use adaptive tetrahedral mesh with minimum element size of 8 microns near each rigid surface, such that the boundary layers are well resolved.

Microsensor measurements and microprofile analysis. Oxygen concentration microprofiles were measured with a fast-response Clark-type O_2 microsensor (tip diameter 10 µm, OX-10, Unisense A/S, Aarhus, Denmark), with steps of 50 µm. The microelectrode was calibrated in air-saturated water and anoxic sodium ascorbate solution. The microsensor was vertically mounted on a motorized micromanipulator (Unisense A/S) and connected to a microsensor multimeter (Unisense A/S). Data acquisition and micromanipulator positioning were controlled by a dedicated software (Sensor TracePro, Unisense A/S). The positioning the tip of the microsensor was monitored with an endoscope. Profiles were taken in light and dark, with a lag period of 1 hour after changing light condition, between 15 and 19 days of growth, only for flume II. Difference of oxygen concentration in the light and dark were calculated for each point and used as a measure of diel variability in oxygen micro-niches. The pooled oxygen concentration distributions (within the biofilm) under light, dark or their difference were significantly not normal (Shapiro test, p-value < 0.01 for each), and they differed significantly for the two morphotypes (Mann–Whitney test, p-value < 0.01 for each). We used the robust Brown-Forsythe Levene-type test from the *lawstat* R package, to test for differences in variance of FFM and SFM. We compared oxygen concentration distributions at comparable depth within the biofilm in the SFM and FFM, in both light conditions, using the unpaired samples t-test with uneven variances. For this analysis, measurement points were pooled in steps of 0.1 mm depth, to account for the uncertainty in the position of the biofilm surface. Distance from clump walls was calculated using the Euclidean distance transform function (from the *scipy.ndimage* package) applied to the binarized DEM. The ratio of the oxygen gradient below the biofilm surface (between 0.05 mm and 0.2 mm) in light and dark (for each profile) was used as an estimate of the relative contribution of photosynthesis and respiration to the net oxygen flux in that point. Also in this case, we used a Welch's two-samples t-test to test for differences between the SFM and FFM.

DNA extraction, sequencing and bioinformatics. We sampled biofilms at the two extremes of the morphological gradient using sterile swabs. Samples were flash frozen at - 80°C and subsequently DNA was isolated using the DNeasy Power Soil kit (QIAGEN). The 16S and 18S rRNA genes were amplified using PCR with the 341f (5’CCTACGGGNGGCWGCAG-3′) and 785r (5′-GACTACHVGGGTATCTAAKCC-3′; Klindworth et al. 2012) and TAReuk454FWD1 (5’-TCGTCGGCAGCGTCAGATGTGTATAAGAGACAG-3’) and TAReukREV3 (5’-GTCTCGTGGGCTCGGAGATGTGTATAAGAGACA-3’; Stoeck et al., 2010) primer pairs, for prokaryotic and eukaryotic community members, respectively. Sequencing libraries were prepared using the Nextera XT kit (Illumina), equimolar pooled and sequenced on a 300 bp paired-end MiSeq (Illumina) run at the Lausanne Genomic Technology Facility (LGTF). Sequencing adapters were clipped from the raw reads which were subsequently denoised and clustered into Amplicon Sequence Variants (ASV) using dada2 (vers. 1.14) (Callahan et al., 2016) as implemented in qiime2 (Bolyen et al., 2019). After taxonomic assignment, autotrophic community members were extracted from the 18S dataset. Analysis of similarity (anosim) was performed on Bray-Curtis distance matrices using the R package *vegan* (Oksanen et al. 2019).

References

Bolyen, E., Rideout, J.R., Dillon, M.R., Bokulich, N.A., Abnet, C.C., Al-Ghalith, G.A., Alexander, H., Alm, E.J., Arumugam, M., Asnicar, F., et al. (2019). Reproducible, interactive, scalable and extensible microbiome data science using QIIME 2. Nat. Biotechnol. *37*, 852–857.

Burliga, A.L., and Kociolek, J.P. (2016). Diatoms (Bacillariophyta) in Rivers. In River Algae, (Cham: Springer International Publishing), pp. 93–128.

Callahan, B.J., McMurdie, P.J., Rosen, M.J., Han, A.W., Johnson, A.J.A., and Holmes, S.P. (2016). DADA2: High-resolution sample inference from Illumina amplicon data. Nat. Methods *13*, 581–583.

Depetris, A., Wiedmer, A., Wagner, M., Schäfer, S., Battin, T.J., and Peter, H. (2019). Automated 3D Optical Coherence Tomography to Elucidate Biofilm Morphogenesis Over Large Spatial Scales. J. Vis. Exp. 1–9.

Hamed, K.H., and Ramachandra Rao, A. (1998). A modified Mann-Kendall trend test for autocorrelated data. J. Hydrol. *204,* 182–196.

Haralick, R.M., Shanmugam, K., and Dinstein, I. (1973). Textural Features for Image Classification. IEEE Trans. Syst. Man. Cybern. *SMC-3*, 610–621.

Menter, F.R. (1993). Zonal two equation kappa-omega turbulence models for aerodynamic flows (Provided by the SAO/NASA Astrophysics Data System).

Muggeo, V.M.R. (2008). segmented: An R package to Fit Regression Models with Broken-Line Relationships. R NEWS *8/1*, 20--25.

Picioreanu, C., van Loosdrecht, M.C.M., and Heijnen, J.J. (1998). Mathematical modeling of biofilm structure with a hybrid differential-discrete cellular automaton approach. Biotechnol. Bioeng. *58*, 101–116.

Pohlon, E., Marxsen, J., and Küsel, K. (2010). Pioneering bacterial and algal communities and potential extracellular enzyme activities of stream biofilms. FEMS Microbiol. Ecol. *71*, 364–373.

R Core Team (2020). R: A Language and Environment for Statistical Computing.

Roeselers, G., Van Loosdrecht, M.C.M., and Muyzer, G. (2007). Heterotrophic pioneers facilitate phototrophic biofilm development. Microb. Ecol. *54*, 578–585.

Schindelin, J., Arganda-Carreras, I., and Frise, E. (2012). Fiji: an open-source platform for biological-image analysis. Nat. Methods.

Sherwood, A.R. (2016). Green Algae (Chlorophyta and Streptophyta) in Rivers. In River Algae, (Cham: Springer International Publishing), pp. 35–63.

Stelzer, R.S., and Lamberti, G.A. (2001). Effects of N : P ratio and total nutrient concentration on stream periphyton community structure, biomass, and elemental composition. Limnol. Oceanogr. *46*, 356–367.

Stoeck, T., Bass, D., Nebel, M., Christen, R., Jones, M.D.M., Breiner, H.-W., and Richards, T.A. (2010). Multiple marker parallel tag environmental DNA sequencing reveals a highly complex eukaryotic community in marine anoxic water. Mol. Ecol. *19*, 21–31.

Taylor, B.W., Keep, C.F., Hall, R.O., Koch, B.J., Tronstad, L.M., Flecker, A.S., and Ulseth, A.J. (2007). Improving the fluorometric ammonium method: Matrix effects, background fluorescence, and standard additions. J. North Am. Benthol. Soc.

Wang, Q., Hamilton, P.B., and Kang, F. (2014). Observations on attachment strategies of periphytic diatoms in changing lotic systems (Ottawa, Canada). Nov. Hedwigia *99*, 239–253.

Oksanen, J., Blanchet, F.G., Friendly, M., Kindt, R., Legendre, P., McGlinn, D., Minchin, P.R., O'hara, R.B., Simpson, G.L., Solymos, P. and Stevens, M.H.H., 2016. vegan: Community Ecology Package. R package version 2.4-3. *Vienna: R Foundation for Statistical Computing.*
